# Predicting Superficial Surgical Site Infections: A Study of the Risk Factors and an Assessment Scale From Western India

**DOI:** 10.7759/cureus.47657

**Published:** 2023-10-25

**Authors:** Dharmendra Shah, Renish Padshala, Shivani R Chaudhary, Shahin Khan, Shashwat Mallik, Giustino Varrassi

**Affiliations:** 1 Department of General Surgery, Medical College Baroda, Vadodara, IND; 2 Department of Pain Medicine, Paolo Procacci Foundation, Rome, ITA

**Keywords:** assessment scale, postoperative complication, general surgery, surgical wound, surgical site infection

## Abstract

Introduction

Superficial surgical site infections (SSSIs) are very common nosocomial infections that can complicate a range of surgeries, resulting in increased morbidity and mortality, and an overall decreased benefit of surgical interventions, along with exorbitant expenditure of healthcare resources. An assessment scale could help in the segregation of the high-risk patient population, and appropriate resources could be directed toward them.

Methods

A prospective observational study was carried out in a tertiary care hospital in Western India with 200 participants. Certain probable preoperative, intraoperative, and postoperative risk factors for SSSIs were assessed for significance of association, and each patient was given a score according to the assessment scale. The predictive power of the scale was calculated.

Results

Body mass index (BMI), preoperative laboratory investigations, and preoperative hospital stay showed a significant association with the complication. Clean-contaminated wounds had a higher incidence of postoperative SSSIs as compared to clean wounds. Postoperatively, fever and the presence of open drains predisposed the patient to complications. The assessment scale was found to have a positive predictive value of 40.94% and a negative predictive value (NPV) of 86.30%.

Conclusion

The factors that could significantly prevent the development of SSSIs are normal preoperative laboratory investigations, less than three days of preoperative hospital stay, and avoiding the use of open drains. The high NPV of the assessment scale means that it can be used as a screening tool to segregate high-risk patients.

## Introduction

Surgical site infections (SSIs) are the third most common hospital-acquired infections having an incidence of around 20% in hospitalized patients, especially plaguing up to 38% of surgical patients in the late 1900s, even in the developed nations [1,2]. A recent shift of preference to minimally invasive surgeries, improved operating theater practices and sterilization methods, and strict guidelines for supervision of the same has resulted in a drop in the incidence of SSIs to around 9.5% in the developed world [3]. Yet, in developing countries, it remains one of the most important preventable complications leading to an increased risk of mortality, re-admission or intensive care unit (ICU) admission, and prolonged hospital stay, thereby causing huge expenditure of healthcare resources. The resultant disability and psychological stresses on the patient and the increased risk-to-benefit ratio of surgical interventions are the other major concerns [4]. There is a disproportionately larger burden of SSIs on the already resource-constrained low-to-middle Human Development Index (HDI) countries [5]. The tropical warm weather (32.4-degree Celsius of average temperature and 62% average humidity) experienced by most of India, across the major part of the year, can also be an added risk factor for SSIs, thereby emphasizing the need for an assessment scale with diagnostic or prognostic value in India [6].

Superficial surgical site infections (SSSIs) are defined as those infections that occur within 30 days of surgical intervention and affect only the skin and subcutaneous tissue at the incision site [7]. While it is fairly common, seen in 5% of all patients undergoing a surgical intervention, it is particularly rampant in those undergoing abdominal surgeries, with up to 40% of patients affected [8,9]. Numerous patient and operative factors have been shown to play a role in the development of SSIs. Some patient-related risk factors include age, nutritional status, coexisting infection, immunodeficient status, and diseases contributing to it, such as diabetes, obesity, and the preoperative length of hospital stay. Factors pertaining to the procedure itself include preoperative shaving and preparation including skin antisepsis, antimicrobial prophylaxis, proper ventilation, disinfection or sterilization of the operating theater and instruments, surgical techniques, drains used, type of dressing, and frequency of cleaning and re-dressing the wound after the operation [4].

Considering these numerous risk factors for the development of SSSIs, it is imperative to evaluate the health status of patients and, subsequently, the risks associated with a surgical intervention. Quantification of the risk of developing an SSSI in the postoperative period is necessary to determine the course of preventive strategies to be used in a patient. Strategies such as prophylactic antimicrobials should not be used in all patients due to concerns of antimicrobial resistance owing to the negligent use of these drugs, and also because the benefits depend inversely on the likelihood of the patient developing an SSI in the first place. Also, an assessment scale that describes an accurate risk model could assist in the comparison of the incidence of SSIs and the associated mortality and morbidity among different healthcare facilities and providers [10].

The present study was conducted to determine the influence of different preoperative, intraoperative, and postoperative factors on the development of SSSIs in a large tertiary hospital in Western India. The predictive power of an assessment scale in identifying patients at risk of developing SSSIs in our geographical region was also assessed [11].

## Materials and methods

We conducted a prospective observational study at a tertiary care center located in Western India to validate an assessment scale for predicting postoperative SSSIs. The study began in April 2019 after receiving permission from the Institutional Ethics Committee for Human Research-Post Graduate Research (IECHR-PGR/ 15-19) and was completed in May 2021. The first 200 patients between 18 and 65 years of age who were admitted to the surgical ward for procedures that could result in clean or clean contaminated sutured surgical wounds over the abdomen were included in the study. Patients aged <18 or >65 years or those with immunocompromised states such as AIDS, chronic renal failure, and diabetes mellitus, and those on chemotherapy were excluded from the study. Patients who underwent non-abdominal surgery or minor surgical procedures such as excision of sebaceous cysts or lipomas or those who did not complete treatment in our hospital were not included in the study. A detailed history was taken and a clinical examination of the patients was conducted; patients were included in the study as per the inclusion criteria. A written informed consent form was obtained from the patients or their relatives prior to their inclusion in the study. This was followed by preoperative laboratory investigations and preparation including preoperative antibiotic prophylaxis at the time of induction (injection of amoxicillin and clavulanic acid 1.2 g for clean wounds and injection of ceftriaxone 1 g and metronidazole 500 mg for clean-contaminated wounds), intraoperative findings, and postoperative findings. All the patients were scored according to the SSSI scoring system by Anwar et al., which included preoperative, intraoperative, and postoperative factors (Appendix) [11].

For body mass index (BMI) calculation, the patient’s body weight and height were measured, and they were classified into ideal weight (18.5-22.9), underweight (<18.5), or overweight (≥23.0). Only the patients undergoing abdominal surgeries such as laparotomy, hernia repair, stoma closure, and pyelolithotomy were included in our study. Since a score of 1 is given to abdominal surgeries, all patients in our study received the same score. Routine investigations such as complete blood count, serum bilirubin, serum creatinine and urea, prothrombin time, and international normalized ratio were performed. Wounds following herniorrhaphy and nephrolithotomy were considered clean wounds, while stoma closure and surgeries involving the gallbladder, biliary tract, and pancreas were grouped as clean contaminated wounds. Postoperatively, body temperature was calculated by taking the mean of the temperatures measured every 6 hours for 48 hours. The drain was removed when the output was <20 cc/day serous discharge.

Patients with a score of ≥11 are considered at risk of developing surgical wound infections, while those with a score <11 are considered to have a lower risk [11]. During postoperative care, 625 mg of amoxicillin and clavulanic acid three times daily for three days was given for clean wounds after allowance of oral intake. Patients with clean-contaminated wounds were given the same preoperative regime by IV route for five days. The wound was inspected for the presence of redness or discharge on completing antibiotics. Swabs from the wound were sent for culture and sensitivity. Patients with a positive culture were given the appropriate antimicrobials based on sensitivity.

The chi-square test or Fisher’s exact test was used to find the significance of study parameters on a categorical scale between two or more groups, and a p-value of <0.05 was considered significant. MedCalc Software Version 12.5.0 was used for the analysis of the data, and Microsoft Word and Excel were used to generate graphs and tables. Receiver operating characteristic (ROC) curve analysis was done to detect the diagnostic accuracy of the total score for SSSIs. Logistic regression with the maximum likelihood estimation method was carried out to determine the predictive power of the total scale to assess SSSIs.

## Results

In our study, 62 (31%) out of 200 patients were found to have developed SSSIs, and the incidence was found to be highest in the age group of 40-49 years (n=18, 29.03%) and lowest in the age group of 20-29 years (n=4, 6.4%), with 24.1% (n=15) in the age group of 30-39 years and 40.32% (n=25) in the age group of 50-65 years. The association of age with the development of SSSIs did not reveal any significance (p=0.150). Only 47 (28.65%) out of 164 non-smokers, and 15 (41.66%) out of 36 smokers developed SSSIs, and, hence, the association of smoking was insignificant (p=0.126). The complications developed least in patients with ideal weight (n=4, 11.4%) and most in overweight patients (n=55, 36.9%) (p=0.007). Preoperative corticosteroids were given to eight patients, of whom one (12.5%) patient developed an SSSI (p=0.248). In our study, all the patients received preoperative prophylactic antibiotics, and only abdominal surgeries were included in the study; hence, we were unable to find their influence. Overall, 25 (36.23%) patients out of 69 having comorbidities (22 had anemia and 47 had hypertension) developed SSSIs. However, neither comorbidity showed a significant association with the development of SSSIs (p=0.245). The development of SSSIs was found to be significantly lower in patients with normal preoperative laboratory findings compared to those with abnormal findings (p=0.0001). Patients who stayed for three or more days (n=40, 42.5%) preoperatively in the hospital developed more SSSIs than those who stayed for less than three days (n=22, 20.7%) (p=0.0015). Also, 28.9% (n=44) of patients with immediate preoperative shaving and 37.5% (n=18) of patients who had shaving the night before surgery developed SSSIs (p=0.264); moreover, 38.7% (n=12) of patients who were given spinal anesthesia and 29.58% (n=50) of patients who were given general anesthesia developed SSSIs (p=0.312) (Table 1). Out of 97 patients who had clean wounds, 23.71% (n=23) developed SSSIs, which was significantly lower than 37.86% (n=39) of patients with clean-contaminated wounds who developed the same (p=0.0305). Patients with normal postoperative body temperature had significantly lower (n=47, 26.1%) development of SSSIs than patients with elevated postoperative body temperature (n=15, 75%) (p=0.00001) (Table 1).

**Table 1:** Association of SSSI with risk factors BMI, body mass index; SSSI, superficial surgical site infections The data are represented in numbers (n) and percentages (%). A p-value of <0.05 has been considered significant.

Risk Factors		SSSI		P-value
		Yes (N=62)	No (N=138)	
BMI	Ideal	4 (11.42%)	31 (88.57%)	0.007
	Underweight	3 (18.75%)	13 (81.25%)	
	Overweight	55 (36.91%)	94 (63.08%)	
Preoperative laboratory findings	Normal	15 (12.19%)	108 (87.80%)	0.000
	Abnormal	47 (61.03%)	30 (38.96%)	
Preoperative hospital stay	1 day	22 (21.56%)	80 (78.43%)	0.001
	2 days	0 (0%)	4 (100%)	
	3 or more days	40 (42.55%)	54 (57.44%)	
Classification of surgical wound	Clean	23 (23.71%)	74 (76.28%)	0.030
	Clean-contaminated	39 (37.86%)	64 (62.13%)	
Postoperative body temperature	Normal	47 (26.11%)	133 (73.88%)	0.000
	Abnormal	15 (75%)	5 (25%)	
Types of drains	Absent	18 (20.68%)	69 (79.31%)	0.007
	Closed	11 (29.72%)	26 (70.27%)	
	Open	33 (43.42%)	43 (56.57%)	

There was a significant association between the presence of drains and the development of SSSIs (p=0.0073). Also, the incidence of SSSIs was 50% (n=12) in patients whose drains were removed one day after surgery and 35% (n=28) in patients whose drains were removed after three days (p=0.195). All patients were discharged after three or more days of surgery, and, hence, no association was found. The use of different suture materials showed no significant association as 31.9% (n=61) of patients with non-absorbable sutures and 11.11% (n=1) of patients with absorbable sutures developed SSSIs (p=0.186). The culture profile of the SSSI patients revealed that the most common organism was *Staphylococcus aureus* (n=28, 45.16%) (Table 2).

**Table 2:** Culture profile of SSSI patients SSSI, superficial surgical site infections The data are represented in numbers (n) and percentages (%)

Causative Organisms	Number of Patients
*Staphylococcus aureus*	28 (45.16%)
*Staphylococcus epidermidis*	13 (20.96%)
*Staphylococcus aureus + Klebsiella pneumoniae*	10 (16.12%)
*Acinetobacter*	8 (12.90%)
*Escherichia coli*	3 (4.83%)
Total	62

**Figure 1:**
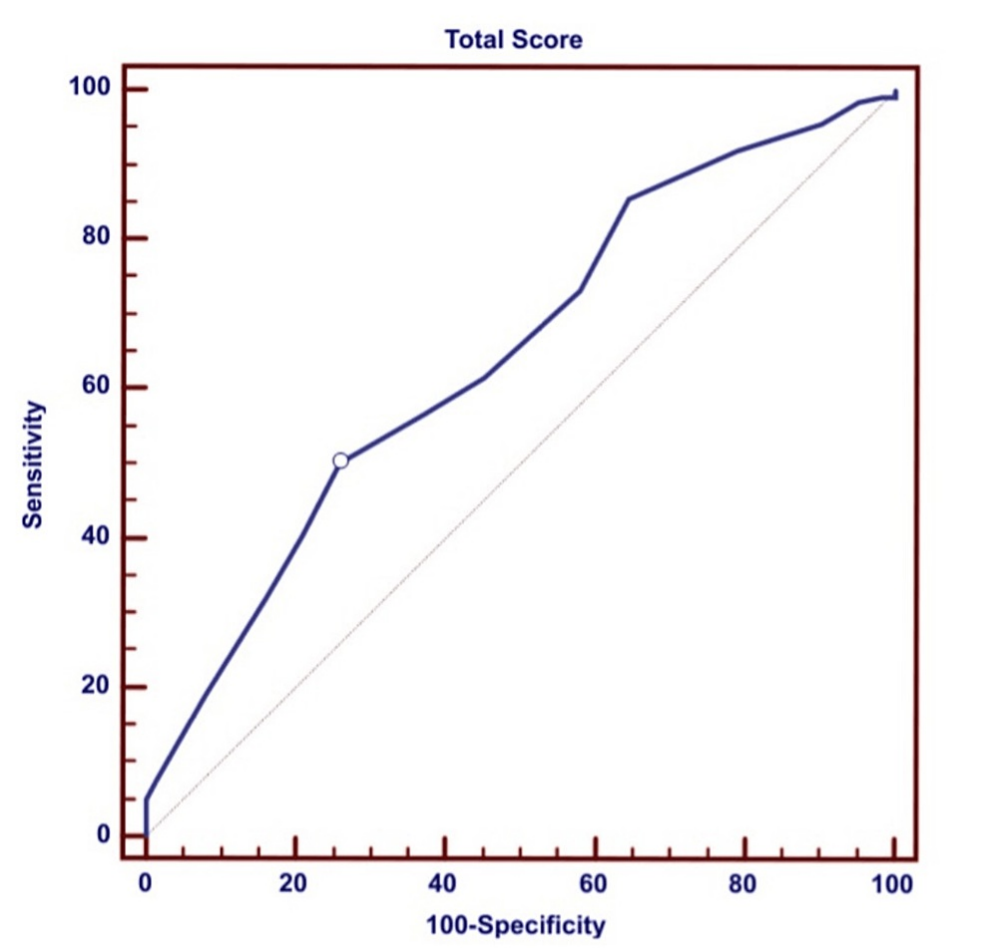
ROC curve ROC, receiver operating characteristic

From the observed data, we plotted an ROC curve analysis for the total score of screening of SSSIs (Figure 1). Diagonal segments are produced by ties. In our study, 127 patients were found to have a score of ≥11, out of which 52 (40.94%) developed SSSI, while only 10 (13.69%) patients who developed SSSI had a score of <11. The positive predictive value (PPV) of the assessment scale was 40.94%, and the negative predictive value (NPV) was 86.30% (Table 3).

**Table 3:** Sensitivity, specificity, PPV, and NPV of the assessment scale SSSI, superficial surgical site infection; PPV, positive predictive value; NPV, negative predictive value PPV is 40.94%, NPV is 86.30%, accuracy is 57.5%, sensitivity is 83.87%, and specificity is 45.65%. The data are represented in numbers (n) and percentages (%)

SSSI Scoring System	SSSI		Total
	Yes	No	
≥11	52 (40.94%)	75 (59.05%)	127
<11	10 (13.69%)	63 (86.30%)	73
Total	62	138	200

## Discussion

Since SSSIs affect a large proportion of surgical patients, subsequently leading to patient distress, decreased benefit of surgical interventions, and an exorbitant increase in healthcare costs, the present study analyzed the influence of various factors in the development of complications in a low-resource setting [4]. The accuracy of the developed assessment scale for patients at risk of SSI was also assessed.

Increased BMI is found to be a major risk factor, with 36.91% (n=55) of overweight patients developing SSSIs, and the same (36.6% of overweight patients) was reported by Anwar et al. [11]. Other studies also reported similar results [12,13]; 61.03% (n=47) of patients with abnormal preoperative laboratory investigations and 12.19% (n=15) of patients with normal preoperative laboratory investigations developed an SSSI in our study, as opposed to Anwar et al.’s study, where only 4.8% with abnormal preoperative laboratory investigations and 34.9% with normal laboratory investigations developed the complication [11]. A preoperative hospital stay of three or more days was associated with a higher SSSI incidence, which is in concordance with other studies [11-14]. Intraoperatively, our study revealed a significantly increased risk of SSSIs with clean-contaminated wounds as compared to clean wounds (n=39, 37.86% v/s n=23, 23.71%), which is backed up by studies by Anwar et al. (38.2% v/s 23.4%) and Bibi et al. (2.5% v/s 1.5%), while the study by Razavi et al. reported opposing results (4.2% v/s 4.6%) [11,12,15]. The incidence of SSSIs also increased from 26.11% (n=47) in patients with normal postoperative body temperature to 75% (n=15) in patients with fever, which is comparable to other studies [11]. In our study, SSSIs were found in 20.69% (n=18) of patients without a drain, 29.73% (n=11) of patients with a closed drain, and 43.42% (n=33) of patients with an open drain. This is in agreement with Anwar et al.’s results, in which SSSIs were found in 29.6% of patients without a drain, 30.4% of patients with a closed drain, and 36.4% of patients with an open drain. There is a significantly lower occurrence of SSSIs in patients without drains and the highest incidence was in patients having open drains (p=0.007). Similar to other studies, the time of drain removal, though, had no association with the development of this complication [11].

SSSIs were more common in the age group of 40-49 years in our study, similar to other studies, while Pathak et al.’s study reported opposing results with an increased incidence in the younger ages [11,13,16,17]. Certain chronic conditions leading to immunodeficient status and delayed wound healing are more common in older ages, which explains the incidence of SSIs in the age group of 40-50 years. In accordance with another study, there was no significant association of the development of SSSIs with smoking status, preoperative corticosteroid administration, or associated anemia or hypertension [11]. The existing literature suggests no significant influence of the time of shaving or the type of anesthesia in the development of SSSIs [11,12]. While the influence of postoperative hospital stay couldn’t be assessed in our study as all patients were discharged after the third postoperative day, Anwar et al.’s study found SSSIs to be prevalent in 34.5% of patients with one-day postoperative hospital stay, 18.2% of patients with a two-day postoperative hospital stay, and 42% of patients with three or more days of postoperative stay [11]. There is no significant influence on the type of suture used for skin closure. This is supported by the findings of Anwar et al., where the incidence was 31.5% for non-absorbable sutures and 14.3% for absorbable sutures, and the findings of Mawalla et al., where the incidence was 15.4% for non-absorbable sutures and 16.9% for absorbable sutures [11,18]. The assessment scale had a PPV of 40.94% and an NPV of 86.30%, similar to the parent study that created it [11]. High NPV means that patients are most likely to not develop an SSSI if the assessment scale score is low.

The study has a relatively small sample size, and, hence, studies on a larger population are required to confirm the generalizability of the results. Since different surgical teams were operating on different cases, the standard of sterility may have been compromised. Patients in immunocompromised states such as AIDS, chronic renal failure, and diabetes mellitus, as well as patients on chemotherapy could have an increased risk of SSSI, but they were excluded from this study, and, hence, their association cannot be determined. The nutritional status was not considered, and malnourishment could predispose to infections, including SSSIs. Additionally, only abdominal surgeries were included, all patients received preoperative antibiotics, and patients were discharged after 48 hours. Thus, we cannot find a relationship between these factors and the development of SSSIs.

## Conclusions

The study concludes that the modifiable risk factors that could be controlled to prevent the development of SSSIs include abnormal preoperative laboratory investigations (total leukocyte count, hemoglobin, serum albumin, coagulation profile, prothrombin activity, and INR), three or more days of preoperative hospital stay, and the use of open drains. By giving scores to surgical patients according to the assessment scale, high-risk patients can be differentiated from low-risk patients. In patients with scores ≥11, the probability of developing SSSIs postoperatively was 40.94%, and in patients with scores <11, the probability of not developing SSSIs postoperatively was 86.30%. While its diagnostic value is limited, patients with low scores are less likely to develop SSSI as the scale has a high NPV, and, hence, it can be used as a screening tool to determine subsequent investigations and prophylactic management. This could lead to the proper allocation of preventive strategies to specific patients and an overall reduction in healthcare costs, which is a boon for developing countries like India.

## Appendices

**Table 4:** SSSI Scoring System [11] SSSI, superficial surgical site infection; BMI, body mass index

Risk Factors	Scores
Preoperative risk factors	
Age (years)	
20-29	0
30-39	1
40-49	2
50-65	3
Smoking	
Non-smoker	0
Smoker	1
BMI	
Ideal (18.5-25)	0
Underweight (<18)	1
Overweight (>25)	2
Preoperative medications	
No corticosteroids given	0
Corticosteroids given	1
Antibiotic prophylaxis given	0
No antibiotic prophylaxis given	1
Site of surgical operation	
Non-abdominal	0
Abdominal	1
Associated diseases (diabetes mellitus/hypertension/cardiac disease/renal disease/HIV/hepatitis B	
No	0
Yes	1
Result of laboratory investigations	
Normal	0
Abnormal	1
Length of hospital stay (days)	
1	0
2	1
3 or more	2
Surgical site preparation (shaving time)	
Immediate preoperative	0
The night before surgery	1
Intraoperative risk factors	
Type of anesthesia	
Spinal	0
General	1
Classification of surgical wound	
Clean	0
Clean-contaminated	1
Contaminated	2
Infected	3
Postoperative risk factors	
Body temperature (97°F-99°F)	
Normal	0
Abnormal	1
Type of drain	
Absent	0
Closed	1
Open	2
Day of drain removal	
1	0
2	1
3 or more than 3 days	2
Length of hospital stay (days)	
1	0
2	1
3 or more	2
Type of suture	
Removable	0
Absorbable	1
Total preoperative score	14
Total intraoperative score	4
Total postoperative score	8
Total score	26
